# Depression in Pulmonary Hypertension: A Systematic Review of Clinical Outcomes, Treatment Interactions, and Emerging Technologies

**DOI:** 10.3390/jcm14030982

**Published:** 2025-02-04

**Authors:** Mira Kramer, Stephan Rosenkranz, Tilmann Kramer

**Affiliations:** 1 Department of Anesthesiology and Intensive Care Medicine, Cologne-Merheim Medical Center, University of Witten Herdecke, Ostmerheimer Str. 200, D-51109 Cologne, Germany; dr.mira.kramer@gmail.com; 2 Clinic III for Internal Medicine, Heart Center of the University of Cologne, Kerpener Str. 62, D-50937 Cologne, Germany; stephan.rosenkranz@uk-koeln.de

**Keywords:** pulmonary hypertension, depression, treatment interactions, pathophysiological mechanisms, telemedicine, mental health

## Abstract

**Background:** Pulmonary hypertension (PH) is characterized by elevated pulmonary artery pressure, often leading to right heart failure and poor prognosis. Depression, a common comorbidity in PH, significantly affects the quality of life. However, mechanistic insights into the bidirectional relationship between PH and depression and specific treatment interactions remain scarce. **Objective:** The object was to examine the clinical and therapeutic implications of depression in PH, focusing on its impact on patient outcomes and cost efficiency. **Methods:** A systematic review of Medline and Google Scholar was conducted to identify studies on depression in PH, emphasizing evidence-based interventions, clinical outcomes, and cost efficiency. Special consideration was given to potential drug interactions between PH medications and depression treatments and to the role of emerging technologies in managing PH-related depression. **Results:** While depression reduces the quality of life and exacerbates disease severity in PH, depression treatment improves outcomes, particularly when tailored to PH-targeted therapies, considering potential drug–drug interactions. Emerging technologies, including telemedicine, offer innovative approaches to manage depression in PH, enhancing access to care and improving adherence to treatment regimens. **Conclusions:** Depression in PH must no longer be neglected and should be acknowledged as both a critical comorbidity and a significant driver of disease progression. Interdisciplinary treatment approaches are necessary, considering potential pharmacological interactions and incorporating emerging technologies like telemedicine to improve patient care. Further research is needed to assess the long-term outcomes of depression treatment in different groups of PH.

## 1. Introduction

### Pulmonary Hypertension and Depression: A Common and Complex Coexistence

Pulmonary hypertension (PH) is a chronic condition characterized by elevated mean arterial pressure > 20 mmHg in the pulmonary arterial circulation based on hemodynamic assessment by right heart catheterization, frequently leading to right heart failure and a limited outcome [1]. PH encompasses a range of conditions classified into five groups based on underlying pathophysiology and clinical presentation. Among these, pulmonary arterial hypertension (PAH, Group 1) is characterized by pre-capillary PH due to vascular remodeling, endothelial dysfunction, and increased pulmonary vascular resistance. Chronic thromboembolic pulmonary hypertension (CTEPH, Group 4) results from unresolved pulmonary emboli causing persistent vascular obstruction and is potentially curable through pulmonary endarterectomy [1]. Estimates indicate that the global prevalence of PH is approximately 1% of the population, with a notable increase to 10% among individuals aged 65 years and older [2]. Depression is a frequent comorbidity in PH, significantly affecting patient well-being and quality of life; yet, it remains underrecognized and insufficiently addressed in clinical practice [1,3,4,5,6,7,8]. The coexistence of PH and depression presents a significant challenge in clinical management. Depression can reduce patient adherence to treatments, hinder disease management, and may contribute to poorer clinical outcomes [9,10]. Understanding the complex mechanisms linking PH and depression is crucial for developing effective treatment strategies and improving patient outcomes [11,12].

A critical challenge in managing depression in PH patients is the potential for drug–drug interactions between PH-specific therapies and antidepressants. Treatments such as phosphodiesterase-5 inhibitors (PDE5i), endothelin receptor antagonists (ERAs), and prostacyclins, commonly used to manage PAH, may interact with antidepressant medications, complicating treatment regimens and requiring careful medication management [13,14].

In recent years, telemedicine and digital health tools have emerged as promising adjuncts to conventional care, offering innovative ways to monitor mental health, facilitate timely interventions, and enhance treatment adherence [15,16,17,18]. These technologies enable personalized and accessible care, particularly for patients with limited mobility or those living in remote areas. Additionally, telemedicine offers environmental benefits by reducing the need for travel to healthcare facilities, thereby contributing to more sustainable healthcare delivery [19,20,21,22,23,24,25].

The high prevalence of depression in PH patients, along with its detrimental effects on treatment outcomes, calls for an integrated multidisciplinary approach to care. This review examines the clinical and therapeutic implications of depression in PH, focusing on its underlying mechanisms, treatment interactions, and the potential role of telemedicine in improving patient outcomes and optimizing care delivery.

## 2. Objectives

This systematic review evaluates the clinical and therapeutic implications of depression in PH, focusing on the bidirectional mechanisms, outcomes, treatment interactions, and strategies for optimizing patient management. It highlights evidence-based interventions, the integration of emerging technologies such as telemedicine, and potential approaches to enhance healthcare sustainability while reducing the economic burden for both patients and the healthcare system.

## 3. Study Selection

An initial search strategy was developed for PubMed and adapted for Google Scholar to identify relevant studies. The search focused on English-language articles investigating the clinical and therapeutic implications of depression in PH, with an emphasis on patient outcomes, treatment interactions, and the role of emerging technologies such as telemedicine and digital health tools. Our comprehensive computerized literature search included a comprehensive set of MeSH terms and keywords related to PH, PAH, CTEPH, depression, bidirectional mechanisms, pathophysiology, treatment interactions, telemedicine, digital health tools, quality of life, guidelines, medication adherence, chronic disease management, health economics, cost savings, patient-centered care, multidisciplinary care, health economics, and innovative therapeutic approaches. The search was limited to articles published up to January 2025. To ensure a comprehensive approach, additional studies were identified by manually reviewing the reference lists of the relevant review articles. Duplicate records and irrelevant publications were removed during the initial screening process.

The inclusion criteria for studies focused on those addressing depression in PH, particularly in the context of mechanisms, clinical outcomes, or therapeutic strategies. Eligible study types included randomized controlled trials, prospective and retrospective studies, review articles, case reports, statistical reports, and World Health Organization reports. The review included studies focusing on adults diagnosed with PH, including subgroups with PAH and CTEPH. Studies were excluded if they were unavailable in full-text format, contained significant methodological flaws or incomplete data, or did not explicitly address the clinical or therapeutic aspects of depression in PH.

The search and screening process identified 106 results after removing duplicates and irrelevant records. The titles and abstracts were screened independently by two reviewers to identify potentially eligible studies. Full texts of the remaining articles were then reviewed against the inclusion and exclusion criteria. Any discrepancies were resolved through discussion or, if necessary, consultation with an independent third reviewer.

This systematic review adhered to the PRISMA guidelines to ensure transparency and rigor in the methodology. The PRISMA checklist (Supplementary Table S1) was followed throughout the review process, and a detailed PRISMA flow diagram (Supplementary Figure S1) illustrates the study selection process, including the number of studies screened, excluded, and included at each stage.

## 4. Prevalence and Impact of Depression in Pulmonary Hypertension

Numerous studies have consistently demonstrated a higher prevalence of depression among individuals with PH compared to the general population [1,3,4,5,7,8,26,27,28,29]. Notably, the prevalence varies among different subgroups of PH. In patients with PAH, depression is reported in up to 53% of cases, reflecting the profound psychological burden associated with this condition. Similarly, individuals with CTEPH show a comparably high prevalence of depressive symptoms, with rates reaching up to 56%. These findings highlight the significant mental health challenges faced by patients in these distinct subgroups [4,8,30].

The hallmark symptoms of PH, including chronic dyspnea, fatigue, and exercise intolerance, significantly limit daily activities and lead to a progressive decline in the quality of life [1]. The physical limitations imposed by these symptoms often prevent patients from participating in work, social, and recreational activities, which are integral to maintaining a sense of normalcy and emotional well-being. This can result in social isolation, as individuals with PH may feel unable to engage in previously enjoyed activities or maintain meaningful relationships due to their reduced functional capacity. Over time, this isolation contributes to feelings of loneliness and disconnection, further exacerbating psychological distress [31]. The progressive loss of independence due to the disabling nature of PH can profoundly impact patients’ self-perception and identity. Tasks that were once routine, such as walking short distances or performing household chores, can become insurmountable challenges, leading to frustration, a sense of helplessness, and diminished self-esteem. The inability to fulfill expected roles, such as those of a caregiver, employee, or active family member, further compounds the emotional burden and fosters a sense of inadequacy. In addition to these social and psychological consequences, the constant uncertainty surrounding disease progression contributes significantly to anxiety and emotional strain. Patients often live with the looming fear of worsening symptoms, potential hospitalizations, or even death, which can overshadow daily life and make long-term planning seem futile. Frequent medical appointments, diagnostic procedures, and treatment adjustments serve as constant reminders of their condition, perpetuating a cycle of worry and emotional exhaustion. The financial burden associated with PH further amplifies this distress. The high costs of medications, specialized care, and travel to medical facilities often place substantial strain on patients and their families, leading to additional stressors that can worsen depressive states. For many, the need to rely on caregivers or family members for financial and physical support exacerbates feelings of guilt and dependency, creating a multifaceted emotional toll. These combined factors illustrate how the interaction between the physical symptoms of PH and their broader psychosocial implications creates a cycle of declining quality of life and worsening psychological health. Importantly, reduced quality of life is observed across all PH subgroups, further highlighting the pervasive impact of this condition [5,7,29,32,33,34,35,36,37,38,39,40].

Depression in patients with PH is not merely a comorbidity but a condition intricately linked to worse clinical outcomes. It is associated with reduced functional status, particularly diminished exercise capacity as measured by the six-minute walk distance (6MWD), a key prognostic indicator in PH. Research indicates that patients with depression exhibit worse hemodynamic profiles and higher hospitalization rates, reflecting both the direct and indirect consequences of this dual disease burden [3,4,10,28,41]. Interestingly, while depression significantly impacts the clinical status and quality of life, it does not appear to affect the long-term survival [39]. Conversely, the debilitating physical symptoms and life-altering consequences of PH can contribute to the development or exacerbation of depression [31].

## 5. Mechanistic Insights into the Relationship Between Pulmonary Hypertension and Depression

**Figure 1:**
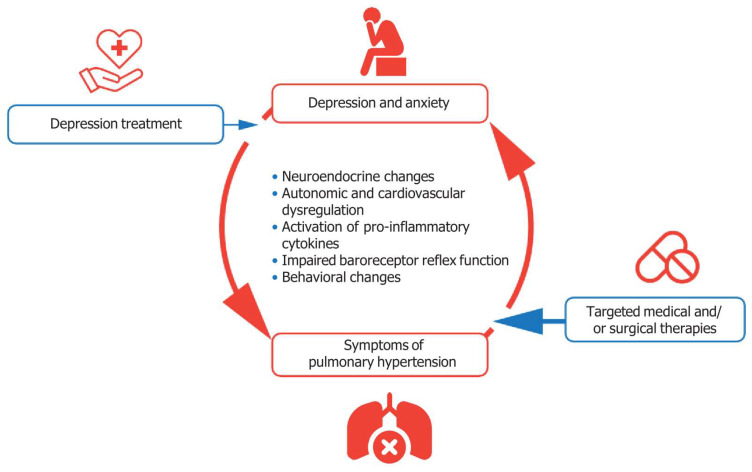
The complex interplay between PH and depression. The bidirectional and mutually reinforcing relationship between PH and depression forms a vicious cycle, exacerbating both conditions. Mechanisms such as neuroendocrine dysregulation, autonomic and cardiovascular dysfunction, systemic inflammation, impaired baroreceptor reflex function, and behavioral changes such as fatigue and reduced physical activity are intricately linked, creating feedback loops that worsen disease severity. These interconnected pathways amplify the burden of each condition, underscoring the need for integrated therapeutic approaches to disrupt this cycle and improve patient outcomes.

The interplay between PH and depression is a complex and multifactorial phenomenon influenced by a variety of interconnected mechanisms, as depicted in Figure 1 [42]. One significant pathway involves neuroendocrine and neurohumoral dysregulation, particularly through the hypothalamic–pituitary–adrenal (HPA) axis. Chronic activation of the HPA axis, commonly observed in stress-related disorders such as depression, results in hypercortisolemia, which negatively impacts cardiovascular health and promotes pulmonary vascular remodeling. Simultaneously, the renin–angiotensin–aldosterone system (RAAS) is activated, contributing to vascular inflammation, oxidative stress, and endothelial dysfunction, which exacerbate the pathophysiology of PH. These neuroendocrine alterations also impair mood regulation, perpetuating depressive states in affected individuals. Autonomic and cardiovascular dysregulation also play a critical role in the relationship between PH and depression. Increased sympathetic nervous system activity, a hallmark of both conditions, leads to heightened sympathetic drive and reduced parasympathetic modulation. This imbalance exacerbates cardiac stress, alters electrophysiological properties of the heart, and predisposes patients to arrhythmias. Additionally, depressed baroreceptor reflex sensitivity compounds this dysregulation, impairing blood pressure stability and contributing to the progression of PH. Inflammatory processes represent another shared mechanistic pathway. Chronic activation of pro-inflammatory cytokines, including tumor necrosis factor-alpha (TNF-α), interleukin-1 beta (IL-1β), and interleukin-6 (IL-6), is observed in both PH and depression. These cytokines not only promote vascular inflammation and pulmonary arterial remodeling but also influence central nervous system pathways involved in mood regulation, thereby increasing the risk of depression in individuals with PH. Impaired baroreceptor reflex function, frequently observed in PH, further influences the relationship with depression. Dysfunctional baroreceptor activity leads to reduced feedback mechanisms for blood pressure regulation, increasing cardiovascular instability, which may heighten psychological distress and contribute to depressive symptoms. Behavioral changes, including fatigue and physical inactivity, form a bidirectional feedback loop that exacerbates both conditions. The debilitating physical symptoms of PH, such as dyspnea and exercise intolerance, result in reduced physical activity, which is a well-documented risk factor for depression. Conversely, depression diminishes motivation and energy levels, further reducing activity and worsening the physiological burden of PH. These intertwined mechanisms highlight the complex interplay between PH and depression, with each pathway uniquely contributing to the exacerbation of both conditions [42,43,44,45,46,47,48,49]. Understanding these mechanisms is crucial for the development of integrated therapeutic strategies that address both the physiological and psychological dimensions of PH and depression. By targeting neuroendocrine and inflammatory pathways, enhancing autonomic regulation, and fostering physical activity, these interventions offer substantial potential to mitigate the bidirectional impact of PH and depression [13,14,15,16,17,18,19,20].

## 6. Therapeutic Interactions Between Pulmonary Hypertension and Depression Treatments

Drug–drug interactions (DDIs) represent a significant challenge in the management of PAH or CTEPH, particularly when addressing comorbid conditions such as depression. Antidepressants, the cornerstone of depression therapy, frequently interact with PAH medications via shared metabolic pathways. These interactions are often mediated by the cytochrome P450 (CYP450) enzyme system, which plays a central role in drug metabolism. Dysregulation or inhibition of CYP450 enzymes due to polypharmacy can lead to altered plasma concentrations of PAH therapies. This imbalance may result in subtherapeutic drug levels or increased toxicity, with potentially severe implications for patient outcomes. Understanding the mechanisms underlying these interactions is critical for optimizing therapeutic regimens and minimizing the associated risks [13,14,50].

### 6.1. CYP450 System in PAH and Depression Therapies

The CYP450 enzyme family consists of multiple isoforms responsible for the metabolism of endogenous compounds and drugs. Among these, CYP3A4 is the most abundant isoform, metabolizing approximately 50% of all marketed drugs, including key PAH therapies such as PDE5i and ERAs. Additional isoforms, including CYP2D6, CYP2C9, CYP2C19, CYP2C8, and CYP1A2, also contribute significantly to the metabolism of PAH drugs and antidepressants. The variability in CYP enzyme activity, caused by genetic polymorphisms, environmental factors, or drug interactions, further complicates the management of patients requiring concurrent therapies for PAH and depression [51].

### 6.2. CYP3A4 and Its Role in PAH Therapy

CYP3A4 is particularly relevant for PAH management due to its involvement in the metabolism of PDE5 inhibitors like sildenafil and tadalafil, as well as ERAs such as bosentan. Inhibition of CYP3A4 by drugs such as fluvoxamine or fluoxetine can elevate plasma concentrations of these therapies, increasing the risk of dose-dependent side effects, including hypotension, headache, and priapism. For example, elevated sildenafil levels have been associated with an increased incidence of cardiovascular side effects, which can exacerbate the hemodynamic burden in PAH patients [14,52].

Conversely, CYP3A4 inducers such as rifampin and carbamazepine accelerate the metabolism of PDE5 inhibitors, reducing their efficacy and potentially compromising the hemodynamic benefits essential for PAH management. Similarly, bosentan, which acts as both a substrate and an inducer of CYP3A4, presents unique challenges. When co-administered with CYP3A4 inhibitors, bosentan plasma concentrations increase, raising the risk of hepatotoxicity. On the other hand, bosentan-induced CYP3A4 activity can reduce plasma levels of co-administered CYP3A4 substrates, necessitating careful dose adjustments and vigilant monitoring to maintain therapeutic efficacy [50,53].

### 6.3. CYP2D6: A Target of Potent Inhibition by Antidepressants

CYP2D6 is a highly polymorphic enzyme involved in the metabolism of numerous drugs, including many antidepressants and certain PAH therapies. Selective serotonin reuptake inhibitors (SSRIs) such as fluoxetine, paroxetine, and fluvoxamine are well-documented inhibitors of CYP2D6. Their co-administration with CYP2D6 substrates can significantly elevate plasma drug concentrations, increasing the risk of adverse effects or therapeutic failure. For instance, tricyclic antidepressants, extensively metabolized via CYP2D6, exhibit increased plasma levels when combined with SSRIs. Such interactions necessitate dose adjustments or the selection of alternative antidepressants to avoid toxicity [54].

CYP2D6 inhibition also has implications for the metabolism of prostacyclin analogues used in PAH therapy. Drugs such as treprostinil, which rely on CYP2D6 for partial metabolism, may accumulate to toxic levels in the presence of potent CYP2D6 inhibitors. This interaction highlights the importance of careful therapeutic monitoring and the need for individualized treatment strategies [14,50].

### 6.4. CYP2C8 and Prostacyclin Analogues

CYP2C8 plays a critical role in the metabolism of prostacyclin analogues, including treprostinil and selexipag. Inhibition of CYP2C8 by antidepressants such as fluoxetine can significantly alter the pharmacokinetics of these drugs, leading to elevated plasma levels and an increased risk of side effects, including flushing, headache, and gastrointestinal discomfort. The interaction between CYP2C8 inhibitors and prostacyclin analogues underscores the need for dose modifications and close monitoring to ensure safety and efficacy [55].

### 6.5. Non-CYP Mediated Interactions in PAH Therapy

In addition to CYP-mediated pathways, non-CYP mechanisms such as drug transport via organic anion-transporting polypeptides (OATP) play an important role in PAH drug pharmacokinetics. Bosentan, a substrate for OATP transporters, demonstrates increased plasma concentrations when co-administered with OATP inhibitors such as fluoxetine. This interaction may elevate the risk of hepatotoxicity and requires dose adjustments to maintain a favorable risk-benefit profile [56]. Furthermore, bosentan’s dual role as an OATP substrate and CYP3A4 inducer adds complexity to its use in polypharmacy settings, particularly when combined with PDE5 inhibitors or other PAH therapies [50].

### 6.6. Antidepressant Selection in PAH Patients

The selection of antidepressants in PAH patients with comorbid depression requires careful consideration of their interaction potential. SSRIs such as citalopram and escitalopram, which exhibit weak CYP450 inhibition, are generally preferred due to their lower DDI risk. In contrast, fluoxetine, paroxetine, and fluvoxamine, which strongly inhibit CYP2D6 and CYP3A4, pose a higher risk of altering PAH therapy pharmacokinetics and require cautious use. Alternative antidepressants such as mirtazapine, which involves minimal CYP-mediated metabolism, may offer a safer profile for patients with refractory depression or those at high risk of DDIs [13,55].

Serotonin–norepinephrine reuptake inhibitors (SNRIs) like venlafaxine and duloxetine, while presenting a moderate DDI risk, are often better tolerated than SSRIs with high inhibitory potential. Individualized dosing strategies and regular monitoring of drug levels are essential to optimize therapeutic efficacy and minimize the risk of adverse effects [50].

### 6.7. Implications for Clinical Practice

The management of PAH in patients with comorbid depression is inherently complex due to the high potential for DDIs mediated by CYP isoforms and transport mechanisms such as OATP. The interplay of CYP inhibition or induction significantly alters the pharmacokinetics of PAH therapies, emphasizing the need for personalized treatment plans. Close therapeutic monitoring, tailored dosing, and the selection of antidepressants with minimal interaction potential are crucial components of effective management.

## 7. Strategies for Optimizing Mental Health Management in Pulmonary Hypertension Care

The integration of mental health services into PH care is critical for improving patient outcomes, particularly for individuals with coexisting depression. Mental health influences treatment adherence, resilience, and the ability of patients to actively manage their condition, which are all essential for successful PH management. Studies highlight the importance of social support networks and community engagement in enhancing psychological well-being. These elements not only improve resilience but also foster a sense of stability and empowerment, which is vital for long-term disease management [57,58,59,60,61,62].

A study by Lynn et al. (2023) included the perspective of a patient living with PAH, emphasizing the need for holistic approaches to mental healthcare [63]. Despite significant advancements in treatment methods that have improved outcomes in PAH [64,65,66,67], mental health outcomes in PH have not shown similar improvements [6]. Therefore, adopting a more focused approach for interventions to treat depression and anxiety in patients with PH holds significant potential for enhancing mental and physical well-being, leading to improved quality of life [8,28,68,69,70] and potentially reduced healthcare demands through reduced symptom severity for pain, anxiety, fatigue, sleep, and dyspnea [70].

Furthermore, a pilot study conducted by Matura et al. (2017) revealed not only improved symptoms but also reduced levels of interleukin-6 after an 8-week slow breathing therapy in women diagnosed with PAH [71]. Slow and controlled breathing has been shown to enhance vagal activity, reduce sympathoexcitation, and positively impact symptoms and prognostic variables [8,72,73,74,75]. Additionally, it has a favorable psychological effect [8,72]. In this context, yoga-based interventions have demonstrated efficacy in reducing symptoms of anxiety and depression, offering a promising complementary approach to support mental health in PH care [76].

Promoting self-management and patient empowerment aligns with broader sustainability goals by optimizing healthcare delivery and reducing the long-term burden on the system. Resilience-building strategies, such as patient education, peer support programs, and community-based interventions, have demonstrated effectiveness in enhancing mental health outcomes. These initiatives can support patients in coping with the psychological challenges of living with depression, fostering improved emotional well-being and mental resilience [57,58,59,60,61,62].

## 8. Emerging Technologies for Mental Health Management in Pulmonary Hypertension

Emerging technologies, including telemedicine, eHealth, and mHealth solutions, offer significant potential for improving the mental health management of patients with PH. Telemedicine, which expanded rapidly during the COVID-19 pandemic [19,77], has demonstrated the ability to enhance access to care, improve adherence to treatment regimens, and reduce hospitalization rates for patients with PH and coexisting depression [78,79,80,81]. By enabling remote monitoring and timely interventions, telemedicine minimizes disruptions in care delivery and provides a valuable tool for addressing the dual challenges of PH and depression [6,31]. Moreover, telemedicine has been shown to reduce transportation-related burdens, with studies indicating that the CO_2_ emissions from telemedicine systems themselves are negligible compared to the significant savings achieved through reduced travel [20].

The World Health Organization (WHO) defines telemedicine as the delivery of healthcare services by healthcare professionals using information and communication technologies for diagnosis, treatment, prevention, and evaluation of diseases and injuries [17,21,82]. Telemedicine includes video consultations, telephone calls, websites, and mobile applications, which collectively enhance the continuity of care while minimizing the risks associated with in-person visits [21]. Driven by the COVID-19 pandemic, the potential of telemedicine in supporting PH management has been demonstrated through remote consultations, virtual follow-ups, and comprehensive disease management, including risk assessment and treatment titration [78,79,80,81]. For patients with depression, telemedicine has shown effectiveness in alleviating depressive symptoms and improving quality of life, even in acute care settings [15,83,84,85]. Furthermore, telemedicine facilitates improved medication adherence in depression management, leading to reduced hospitalization rates and better long-term outcomes for PH patients [16,86,87,88].

Electronic health (eHealth), encompassing a range of informatics applications for healthcare management, provides additional tools to enhance patient care [89,90]. eHealth enables remote monitoring, allowing for early symptom identification and timely responses to exacerbations, while also empowering patients through self-management tools [90,91,92,93]. These technologies shift the balance of responsibility from healthcare professionals to patients, leading to improved health-related quality of life [90,94,95]. Mobile health (mHealth), a subset of eHealth utilizing mobile devices [90,96], has also been recognized for its role in managing chronic conditions, including PH [90,97,98]. Although evidence on eHealth use in PH patients is limited and primarily focuses on validating technology tools, its potential benefits for disease management remain significant [90,98].

In a recent study by Stubbs et al. (2022), patients with PH successfully performed exercise capacity tests at home and completed quality of life questionnaires electronically. These findings highlight the potential of telemedicine strategies to enhance remote monitoring and patient engagement [99]. Additionally, leveraging telehealth platforms for therapy sessions in patients with PH and concomitant depression has been associated with improved symptoms, reduced hospitalizations, and decreased demand for acute in-hospital care. For low-risk PH patients, virtual follow-ups provide a convenient alternative to in-person visits, further reducing logistical challenges and resource utilization [78,79].

Incorporating telemedicine and digital health solutions into routine PH care pathways represents a promising approach to enhance mental health management while addressing the systemic and logistical challenges often associated with traditional care delivery. These innovations offer an opportunity to provide high-quality modern care for PH patients with depression while aligning with broader goals of sustainability and accessibility in healthcare [78,79,80,81].

### Cost Efficiency in Pulmonary Hypertension Care

Recent developments in the treatment of PAH have revolutionized the available options for patients [64,65,66,67]. Current guidelines recommend the use of initial combination therapies [1]. While this approach may incur substantial pharmacy costs, emerging evidence indicates that combination therapy has the potential to reduce hospitalizations, which are a major contributor to the healthcare expenses associated with PAH [100,101]. Since the economic impact of PAH can be substantial, this paradigm shift in treatment strategies not only improves patient outcomes but also offers the possibility of optimizing healthcare costs in the management of PAH.

Depression imposes a substantial economic burden on individuals and contributes to productivity losses and medication noncompliance in patients with PH, ultimately resulting in increased healthcare expenses [6,31]. Implementation of depression treatment not only enhances medical compliance, symptoms, and quality of life [8,28,68,69,70,72,73,74] but also potentially reduces the need for hospitalizations, resulting in significant cost savings for both patients and healthcare systems.

There are several strategies to achieve cost savings in PH care through effective depression treatment. These include integrating mental health services into the PH care pathway, improving access to evidence-based depression treatments, promoting self-management and patient empowerment, and implementing collaborative care models that involve a multidisciplinary team approach [4,6,8,10,27,31]. The adoption of telemedicine for the treatment of depression represents an additional opportunity to reduce costs in the healthcare system. Telemedicine facilitates remote monitoring and timely interventions for depression, thereby reducing the need for in-person visits and associated travel costs, particularly for patients in remote areas [102,103,104,105]. Finally, preventive healthcare approaches demonstrate a higher cost-effectiveness compared to relying solely on reactive measures [106].

## 9. Limitations

This systematic review has limitations related to the heterogeneity in study designs, population characteristics, and the scope of the included studies. The availability of evidence on specific clinical aspects, such as treatment interactions and emerging technologies, remains limited, which may restrict the depth of the conclusions drawn. Additionally, the findings may have limited generalizability to broader populations or clinical settings beyond those studied. The exclusion of non-English-language articles might have led to the omission of relevant research published in other languages. Although this review was not preregistered in a public registry, adherence to PRISMA guidelines ensured a transparent and rigorous methodological approach.

## 10. Conclusions

Effective management of depression in patients with PH is essential for improving quality of life and advancing clinical care. Evidence-based interventions that address the bidirectional relationship between PH and depression, while carefully managing DDIs mediated by cytochrome P450 enzymes, enhance treatment safety and efficacy. Individualized plans that consider the pharmacokinetics of PH therapies and the metabolic effects of antidepressants are vital. Collaborative approaches that integrate expertise from multiple disciplines ensure tailored care for this complex patient population.

Telemedicine strengthens traditional PH and depression management by improving access to timely interventions and enabling remote monitoring. This approach not only enhances adherence to therapy but also supports sustainability by minimizing travel-related emissions.

Future research should focus on developing personalized multimodal therapeutic strategies that integrate innovative technologies and address therapeutic interactions comprehensively, further improving outcomes for patients with PH and coexisting depression.

## Data Availability

Not applicable.

## Supplementary Materials

The following supporting information can be downloaded at https://www.mdpi.com/article/10.3390/jcm14030982/s1, Figure S1: PRISMA 2020 flow diagram for new systematic reviews which included searches of databases and registers only; Table S1: PRISMA 2020 Checklist.
